# Communication interventriculaire post infarctus du myocarde: à propos d’un cas et revue de la litterature

**DOI:** 10.11604/pamj.2017.28.242.12970

**Published:** 2017-11-17

**Authors:** Tahir Nebhani, Said Jidane, Hicham Bakkali, Lahcen Belyamani

**Affiliations:** 1 Service des Urgences Medico-Chirurgicales de l’Hôpital Militaire d’Instruction Mohammed V, Rabat, Maroc

**Keywords:** Infarctus du myocarde, communication interventriculaire, Maroc, Myocardial infarction, inter-ventricular communication, Morocco

## Abstract

La rupture septale secondaire à l'infarctus du myocarde est une complication aiguë redoutable dont la mortalité est non négligeable à la phase précoce. Nous rapportons le cas d'une rupture septale apicale chez un patient de 70 ans admis aux urgences pour prise en charge d'un post infarctus de myocarde antéro-septal. C'est une urgence extrême dont la prise en charge doit être multidisciplinaire. Nous mettons le point sur les facteurs de risque et sur le traitement à proposer pour ce type de complication.

## Abstract (en)

Ventricular septal rupture (VSR) after myocardial infarction is a fearsome acute complication whose mortality is not negligible in the early phase. We report the case of a 70-year old patient with apical septal rupture admitted to the Emergency Department with ventricular septal rupture after anteroseptal myocardial infarction. This disease is of the utmost urgency, so its management should be multidisciplinary. This study aims to focus on the risk factors and the treatment of this complication.

## Introduction

La communication interventriculaire (CIV) post infarctus du myocarde (IDM) est une complication rare mais non exceptionnelle, elle survenait dans 1 à 2% des infarctus avant l'ère de la thrombolyse [1]. Durant les dernières années, cette fréquence a diminué compte tenu de la prise en charge médicale plus agressive diminuant la fréquence à 0.2%, cependant la mortalité reste très élevée [2]. C'est une urgence extrême imposant une prise en charge multidisciplinaire associant les urgentistes, les cardiologues et les chirurgiens cardiovasculaires. Nous rapportons dans cette observation le cas d'un patient qui a présenté un syndrome coronarien aigu avec sus décalage du segment ST compliqué d'une rupture septale.

## Patient et observation

**Figure 1:**
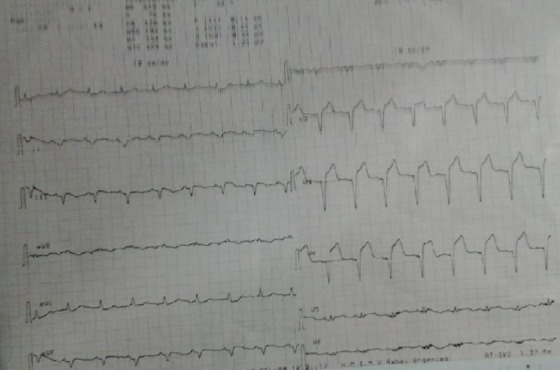
Electrocardiogramme montrant un sus décalage du segment ST dans le territoire antero–septal

**Figure 2:**
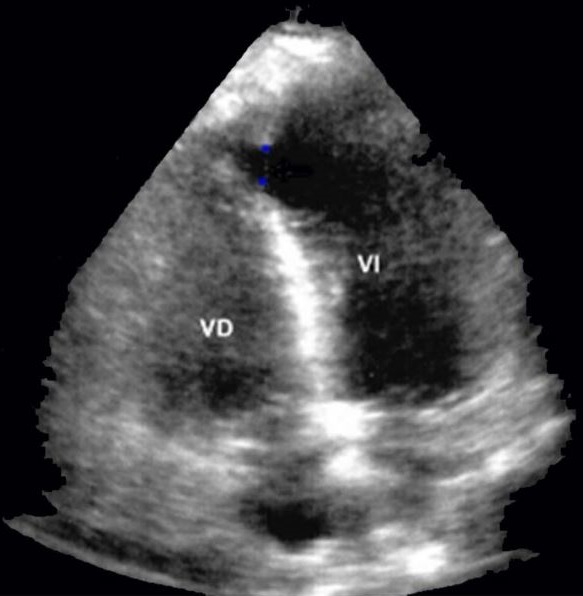
Coupe apicale quatre cavités d’echocardiographie montrant une rupture septale apicale

Il s'agit d'un Homme de 70 ans, ayant comme facteurs de risque cardiovasculaire un tabagisme chronique à raison de 40 paquets année et une dyslipidemie, admis aux urgences pour douleur thoracique. Le début de la symptomatologie remonte à 2 jours par l'installation d'une douleur thoracique infarctoide associée à une dyspnée stade IV de la NYHA évoluant dans un contexte d'apyréxie et d'altération de l'état général. Le patient a été pris en charge initialement en salle de déchoquage dont l'examen clinique trouvait un malade conscient. Sur le plan hemodynamique la fréquence cardiaque était à 99 bpm, la tension artérielle à 75/46 mmHg, avec signes de choc (extrémités froides, cyanosées et des marbrures), on notait la présence des signes d'insuffisance cardiaque droite avec une turgescence des veines jugulaires et un reflux hépato-Jugulaire. L'auscultation cardiaque objectivait un souffle systolique en rayon de roue au foyer mitral. Sur le plan respiratoire le patient était tachypneique à 35 cpm. Sa Saturation pulsée en oxygène était à 94% à l'air libre. L'auscultation pleuro-pulmonaire trouvait des râles crépitants bilatéraux en marrée montante. L'électrocardiogramme avait objectivé un sus-décalage du segment ST dans le territoire antéro-septal (Figure 1). La troponine I cardiaque était élevée à 16.44 ng/ml, la fonction rénale altérée et la protéine C réactive à 200 mg/l. L'échocardiographie transthoracique réalisée au lit du malade avait montré un ventricule gauche non dilaté avec communication interventriculaire apicale de 7mm, la fraction d'éjection était à 57%. Le ventricule droit non dilaté avec fonction systolique conservée (Figure 2). Le diagnostic de CIV post IDM a été retenu. Le patient a été mis sous dobutamine à 10ug/kg/min, sous double anti-agrégants plaquettaires acide. Acétyl-salicylique 250mg en IVD et clopidogrel 600mg en per os et héparine de bas poids moléculaire 60mg toutes les 12 heures en sous cutanées. Puis le patient a été transféré en unité de soins intensifs de cardiologie ou un ballon de contre pulsion aortique a été mis en place en attente d'une chirurgie cardiaque. Les suites étaient sans particularité et le patient a été opéré un mois après.

## Discussion

La rupture septale post-infarctus du myocarde constitue un événement catastrophique pouvant engagé le pronostic vital, dont l'incidence actuellement est de 0.2% avec une mortalité qui reste tout de même élevée dépassant les 90% [2]. Le délai moyen de survenue de cette complication est de 6 (3-9) jours. Dans notre cas le délai de survenu était de 48h après le début du SCA ST+. Les facteurs de risque les plus rapportés sont l'âge avancé, le territoire antérieur de l'infarctus et le sexe féminin [1]. L'IDM peut être compliqué d'une insuffisance ventriculaire droite dans 45% des cas, gauche dans 30% et/ou globale dans 20% des cas. Un choc cardiogénique est décrit dans 60% des cas, comme c'est le cas chez notre patient [2]. Le diagnostic positif de la rupture septale est échocardiographique. Elle est antérieure dans 69% et postérieure dans 31% des cas [3]. Le traitement est médico-chirurgical ainsi qu'interventionnel. Une réparation chirurgicale urgente a été initialement proposée depuis les années 1980, parce que le traitement médical seul a été soldé par une mortalité avoisinant les 100% [4]. L'AHA/ACC recommande une chirurgie réparatrice en urgence; malgré qu'une chirurgie précoce (3 jours-4 semaines) a une mortalité intra-hospitalière de 52%, alors que pour une chirurgie retardée, elle est de l'ordre de 8% [5]. En attente d'une cure chirurgicale un ballon de contre pulsion intra aortique peut être mis en place (60% des cas) [6]. Dans la décade passée, la fermeture percutanée de la CIV avait été émergée comme une alternative prometteuse [7]. Une bonne prise en charge est définie par l'amélioration ou l'élimination du choc cardiogénique relatif au shunt gauche droit, conséquent à la CIV. Pourtant 40% des patients gardent un shunt résiduel, et requièrent une réintervention chirurgicale [6].

## Conclusion

Le pronostic des CIV post IDM est effroyable, la prise en charge chirurgicale de fermeture des communications inter ventriculaires post-infarctus, est recommandée pour améliorer la survie des patients.

## Conflits d’intérêts

Les auteurs ne déclarent aucun conflit d'intérêts.
